# Exploring microbial ecology in public swimming pools: a metagenomic investigation of community structure and environmental correlates

**DOI:** 10.1186/s12866-026-05157-7

**Published:** 2026-05-21

**Authors:** Ling Tong, Yongping Liu, Fengchan Han, Yuanqiang Jiang, Shengjie Ying, Bo Zhang, Yu Cheng, Zhongling Liu, Yewen Shi, Mingjing Xu, Chuanxi Tang, Shaofeng Sui, Tian Chen

**Affiliations:** 1 Department of Environmental Health, Institute for Monitoring and Control of Health Risk Factors, Shanghai Municipal Center for Disease Control and Prevention, Shanghai, 201107 China; 2 Shanghai Putuo District Center for Disease Control and Prevention (Shanghai Putuo District Health Supervision Institute), Shanghai, 200333 China; 3 Shanghai Songjiang District Center for Disease Control and Prevention (Shanghai Songjiang District Health Supervision Institute), Shanghai, 201600 China; 4 Shanghai Minhang District Center for Disease Control and Prevention (Shanghai Minhang District Health Supervision Institute), Shanghai, 201199 China; 5 Shanghai Huangpu District Center for Disease Control and Prevention (Shanghai Huangpu District Health Supervision Institute), Shanghai, 200023 China; 6 Shanghai Xuhui District Center for Disease Control and Prevention (Shanghai Xuhui District Health Supervision Institute), Shanghai, 200237 China; 7 Shanghai Jing’an District Center for Disease Control and Prevention (Shanghai Jing’an District Health Supervision Institute), Shanghai, 200041 China; 8 Shanghai Changning District Center for Disease Control and Prevention (Shanghai Changning District Health Supervision Institute), Shanghai, 200051 China

**Keywords:** Public swimming pools, Pathogenic microorganisms, Metagenomic sequencing, Water quality management

## Abstract

Epidemiological studies have identified correlations between swimming and outbreaks of various infectious diseases. However, a comprehensive understanding of the pathogens present in public swimming pool water has yet to be systematically established. Swimming pool water samples were collected from 20 indoor public swimming pools in Shanghai, China during the summer of 2023. After quality inspection of the extracted nucleic acid, the qualified samples were subjected to metagenomic sequencing to profile the microbial communities of swimming pool water. A total of 24,035 microbial species were identified with the abundance of bacteria (99.46%), followed by archaea (0.29%), viruses (0.20%), and fungi (0.05%), including 441 pathogenic species, 23 of which were classified as biosafety level 3 (BSL-3) microorganisms. Environmental sources constituted the dominant origin (86.00%) of the pool water microbiome. Additionally, suburban pools demonstrated greater microbial diversity than urban pools (*P* < 0.05). The abundance of viruses exhibited a positive correlation with the concentration of urea in pool water (*r* = 0.31, *P* < 0.05). This study demonstrated that swimming pool water serves as a potent reservoir and mixing vessel for various highly pathogenic microorganisms. Effective water quality management strategies are essential to mitigating the potential public health threats of public swimming pools.

## Background

Swimming has become an increasingly popular recreational activity worldwide, offering a combination of fitness, leisure, and social interaction. However, direct exposure to pool water, coupled with the presence of both healthy and unhealthy swimmers, swimming may pose a significant risk of infectious disease transmission if water quality management is inadequate. Epidemiological studies have identified correlations between swimming and outbreaks of various infectious diseases, including acute conjunctivitis, pharyngoconjunctival fever, viral gastroenteritis, hepatitis A, ear infection, and dermatological infections [1–8]. Additionally, parasitic infections have been reported, including *Cryptosporidium* outbreaks in hotel swimming pools in Nagano Prefecture, Japan, and Staffordshire, UK [9, 10].

These incidents highlight the presence of pathogenic microorganisms in swimming pool water. Previous studies have detected *Pseudomonas aeruginosa*, *Salmonella*, *Vibrio*, and *Acanthamoeba* in both pool water and swimming aids, with *P. aeruginosa* being the most frequently identified opportunistic pathogen [11–14]. Additionally, viral contaminants, such as enteroviruses (e.g., poliovirus Sabin 1), adenoviruses, and even non-enteric viruses like papillomaviruses and polyomaviruses, have been detected in swimming pool water [15, 16]. These contaminants are likely introduced via bodily secretions from infected individuals. Furthermore, meta-analyses have revealed the presence of fungal contaminants in swimming pools including dermatophytes (2.78%), yeasts (14.29%), and saprophytes (73.73%) [17]. Several relevant studies have been conducted in China. For example, an investigation in Beijing reported the presence of *Cryptosporidium* oocysts and *Giardia* cysts in swimming pool water, with positivity rates of 16.70% and 15.00%, respectively [18]. Xiaohong Wei et al. detected *Giardia*, *Cryptosporidium*, antibiotic-resistant *Pseudomonas aeruginosa*, total coliforms, and *Escherichia coli* in public swimming pool water in Guangzhou [19]. Additionally, human adenovirus type 7 (HAdV-7) was isolated from a swimming pool in Zhejiang Province, in 2019 [20].

Assessing the risk of pathogenic microorganisms in swimming pool water is imperative for developing effective management strategies and safeguarding public health. Quantitative risk assessments have been conducted for several key pathogens. For instance, Rice et al. evaluated the health risks of *P. aeruginosa* and proposed mitigation strategies, while Suppes et al. estimated the risk of *Cryptosporidium* infection to be 2.6 × 10⁻¹ per swimming event [21, 22]. However, most previous studies have primarily relied on culture-based methods or PCR assays targeting specific microorganisms, limiting the scope of pathogen detection. A limited number of studies have employed sequencing techniques to detect non-tuberculous mycobacteria in public swimming pool water or to compare the efficacy of different disinfection methods [23, 24].

Despite these advances, a comprehensive understanding of the full spectrum of pathogens present in public swimming pool water has yet to be systematically established. Therefore, implementing advanced detection methodologies to systematically identify pathogenic microorganisms in public swimming pool water represents a crucial scientific priority. The metagenome, first conceptualized by Handelsman et al. in 1998, refers to the collective genomes of microbial communities in environmental samples [25]. This approach is particularly valuable for detecting unculturable pathogens and is now indispensable in public health surveillance [26–28]. The application of metagenomic sequencing to the analysis of swimming pool water enables a comprehensive elucidation of microbial diversity and pathogen distribution patterns. This, in turn, provides a crucial basis for controlling pathogenic microorganisms in swimming pool water and enhancing public health protection, particularly in high-density urban environments such as Shanghai.

## Materials and methods

### Selection of swimming pool

**Fig. 1:**
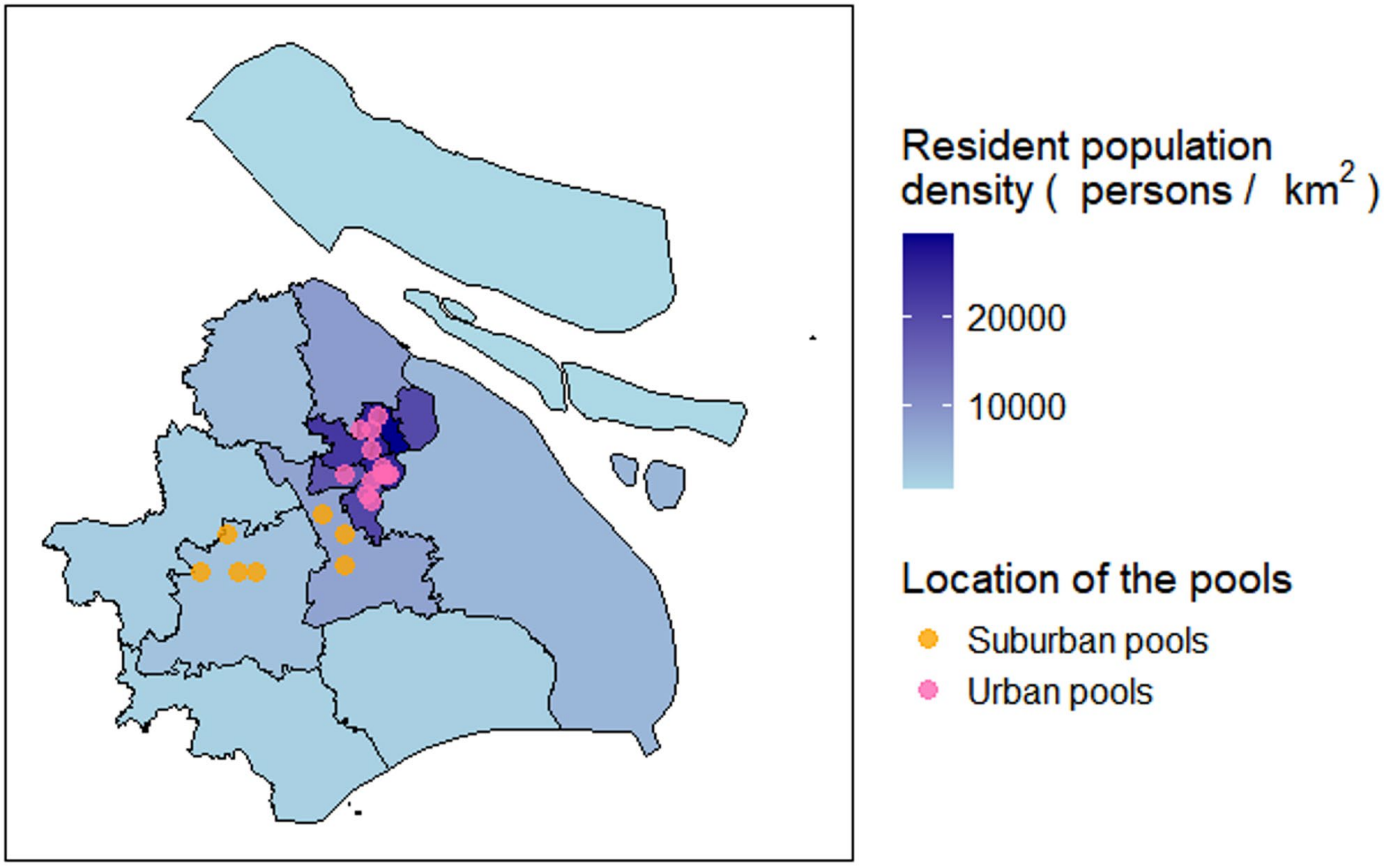
The locations of swimming pool water sampling sites in Shanghai, China

In 2023, field investigations were conducted to collect baseline data from public indoor swimming pools in Shanghai, China. The collected parameters included geographic coordinates, surface area, annual visitor statistics, operational duration, and daily average attendance. Swimming pools were classified by the population density of their administrative district: those in areas with a density greater than 10,000 persons/km^2^ were designated as urban, and those in areas with a density of 10,000 persons/km^2^ or less were designated as suburban. A total of 20 swimming pools were sampled, comprising 7 urban and 13 suburban pools. The locations of swimming pool water sampling sites in Shanghai, China were displayed in Fig. 1. The inclusion criteria for pool selection were as follows: indoor public swimming pool with an operational duration of at least one year, possession of a valid hygiene license, a minimum daily attendance of 20 visitors during the summer, and a willingness to participate in this study. Following this rigorous selection process, a total of twenty public indoor swimming pools were enrolled in this research. The baseline data of enrolled pools are presented in Table 1.

**Table 1:** Baseline data of enrolled swimming pools

Sample ID	Geographic coordinates	Surface area (m^2^)	Operational duration (year)	Summer daily attendance (person/day)
M1	Suburban	375	9	334
M2	Suburban	312	12	157
M3	Suburban	1050	15	212
J1	Urban	500	14	59
J2	Urban	180	18	426
J3	Urban	500	7	145
J4	Urban	375	5	178
H1	Urban	250	15	29
H2	Urban	230	13	121
H3	Urban	385	13	35
H4	Urban	175	23	22
C1	Urban	200	9	36
S1	Suburban	220	7	29
S2	Suburban	375	6	23
S3	Suburban	220	9	34
S4	Suburban	500	3	127
X1	Urban	176	5	68
X2	Urban	240	6	29
X3	Urban	1035	2	1350
X4	Urban	1180	14	1002

### Detection of physicochemical water quality

Water quality parameters, including temperature, pH value, urea concentration, turbidity, residual disinfectant levels, and oxidation-reduction potential (ORP), were measured according to GB/T 18,204 standard (methodological details provided in Table 2) [29, 30]. Sampling followed by a stratified approach: two sampling points designated for pools ≤ 1000 m² and three sampling points for pools > 1000 m². Triplicate measurements were taken at each sampling point and averaged for the final analysis.

**Table 2:** Methods for physicochemical water quality testing in swimming pool water

Physicochemical parameter	Detection method
Temperature	Thermometer
pH value	pH meter
Urea	Spectrophotometer
Turbidity	Scattering turbidity meter
Residual disinfectant	Spectrophotometer
ORP	Potentiometer

### Sampling strategy

Sterile sodium thiosulfate-treated bags were used to collected 5 L samples at a depth of 10 ~ 20 cm below the water surface and at least 30 cm away from the side walls. Sampling was conducted at two points for pools ≤ 1000 m^2^ and at three points for pools > 1000 m^2^. After sampling, all samples were transported under dark conditions at 0 ~ 4 °C and processed within 8 h.

### DNA extraction and library preparation

Samples were concentrated through 0.2 μm filtration (Jinteng Co., China), and total microbial DNA was extracted following the instructions of the DNA extraction kit (QIAamp^®^ Fast DNA Stool Mini Kit, Qiagen, Hilden, Germany). The concentration and purity of DNA were assessed using a NanoDrop 2000 UV spectrophotometer (Thermo Fisher Scientific, Waltham, MA, USA). All DNA samples exhibited a single, clear main band without significant smearing, indicating no obvious degradation by 1% agarose gel electrophoresis. The high integrity of the DNA met the requirements for subsequent high-throughput sequencing and molecular biology experiments. DNA fragmentation was performed using an S220 Focused-ultrasonicator (Covaris, USA), and purification was carried out with Agencourt AMPure XP beads (Beckman Coulter, USA). Library construction was performed using the TruSeq Nano DNA LT Sample Preparation Kit (Illumina, San Diego, CA, USA).

### Metagenomic sequencing

Sequencing was performed on the Illumina Novaseq 6000 platform, generating 150 bp paired-end reads [31]. Raw sequencing data were processed using Fastp (v 0.20.1) to remove adapters, filter out low-quality bases, and eliminate reads containing ambiguous (N) bases [32]. MEGAHIT (v 1.2.9) was employed for metagenomic assembly [33]. Open reading frames (ORFs) were predicted from the assembled scaffolds using Prodigal (v 2.6.3) and subsequently translated into amino acid sequences [34]. MMSeqs2 (v 13.45111) was used to construct a non-redundant gene catalog from the predicted genes across all samples, with clustering parameters set at 95.00% sequence identity and 90.00% coverage [35]. The longest gene in each cluster was selected as the representative sequence. For gene abundance quantification, clean reads of each sample were aligned to the representative sequences using Salmon (v 1.8.0) with 95.00% identity threshold, and gene abundance was calculated based on aligned read counts and gene length [36]. Genes with fewer than 2 reads across all samples were removed to obtain the final non-redundant gene set, which we refer to as the Unigene. Taxonomic annotation of the predicted genes was conducted via DIAMOND BLASTx against the NCBI non-redundant database (NR), applying stringent thresholds requiring an E-value ≤ 1 × 10^− 5^, a minimum sequence identity of 80.00%, and a query coverage of at least 50.00% [37]. The pathogenic microorganisms and their biosafety levels were identified by matching with the Global Catalogue of Pathogens (https://nmdc.cn/gcpathogen).

To avoid false positives from single-gene assignments, especially for BSL-3 pathogens, we applied additional filters. A species was considered present only if (i) at least three independent genes from the non-redundant gene set were assigned to that species, with at least two genes located on different assembled scaffolds; (ii) for BSL-3 species, raw reads mapping to candidate genes were re-aligned to the complete reference genome (BLASTn, ≥ 99% identity, ≥ 100 bp), requiring coverage of at least two distinct genomic regions ≥ 500 bp apart; and (iii) no equally good match existed to another pathogenic species in the same genus.

### Statistical analyses

Statistical analyses and visualizations were performed using R (version 4.1.3). To elucidate the origins of the pool water microbiota, the NCBI BioSample database was queried via the species names corresponding to the 500 highest-abundant microorganisms found in the pool water microbial communities [38]. *α*-diversity analysis was performed using the *MicrobiotaProcess* package (version 1.6.6) [39]. *β*-diversity analysis, including Principal Co-ordinates Analysis (PCoA), was conducted with the *Vegan* package (version 2.6.4) [40]. Linear Discriminant Analysis Effect Size (LEfSe) analysis was conducted using the *Microeco* package (version 0.16.0) [41]. The Mantel test was performed using the *LinkET* package(version 0.0.7.4) [42]. Welch’s *t*-test was used to compare species differences between groups. Spearman correlation analysis was employed for correlation analysis. The significance level was set at *α* = 0.05.

## Results

### Water quality characteristics of swimming pools

Table 3 summarizes the physicochemical characteristics of the swimming pool water samples. The median water temperature was 29.10 ℃ (range: 25.40–31.40 ℃), with 70.00% (14/20) of the samples falling within the range of 23.00–30.00 ℃ required by the Chinese national standard *Hygienic indicators and limits for public places* (GB 37488 − 2019) [43]. The median ORP was 663.30 mV (range: 535.00-806.60 mV), and 75.00% (15/20) of the samples met the required threshold of ≥ 650.00 mV (GB 37488 − 2019). Free residual chlorine concentrations ranged from 0.19 to 7.05 mg/L, where 75.00% (15/20) of the samples complied with the national standard (GB 37488 − 2019) limit of 0.30-1.00 mg/L, 10.00% (2/20) were below 0.30 mg/L and 15.00% (3/20) exceeded 1.00 mg/L. Regarding pH, 75.00% (15/20) of the samples were within the required range of 7.00-7.80 (GB 37488 − 2019). For urea, 85.00% (17/20) of the samples met the standard (GB 37488 − 2019) limit of ≤ 3.50 mg/L. Notably, all samples (100.00%, 20/20) were within the turbidity limit of ≤ 1.00 NTU.

**Table 3:** Statistical description of physicochemical factors in swimming pool water

Factors	Range	$\overline{\mathbf{X}} \boldsymbol{\pm} \boldsymbol{\mathbf{SD}}$	*P*_25_	*P*_50_	*P*_75_	Compliant samples (No.)*	Compliance rate (%.)*
Water temperature (℃)	25.40–31.40	29.00 ± 1.50	28.00	29.10	30.10	14	70.00
ORP(mV)	535.00-806.60	677.60 ± 63.00	641.00	663.30	691.50	15	75.00
Free residual chlorine (mg/L)	0.19–7.05	1.22 ± 1.83	0.49	0.62	0.80	15	75.00
pH	6.49–7.87	7.44 ± 0.31	7.32	7.40	7.61	17	85.00
Urea (mg/L)	0.02–10.53	2.35 ± 2.71	0.47	1.90	2.95	17	85.00
Turbidity (NTU)	0.12–0.88	0.40 ± 0.23	0.22	0.32	0.54	20	100.00

*As specified in the Chinese national standard Hygienic indicators and limits for public places (GB 37488 − 2019)

### Microbial community composition in the pool water

**Fig. 2:**
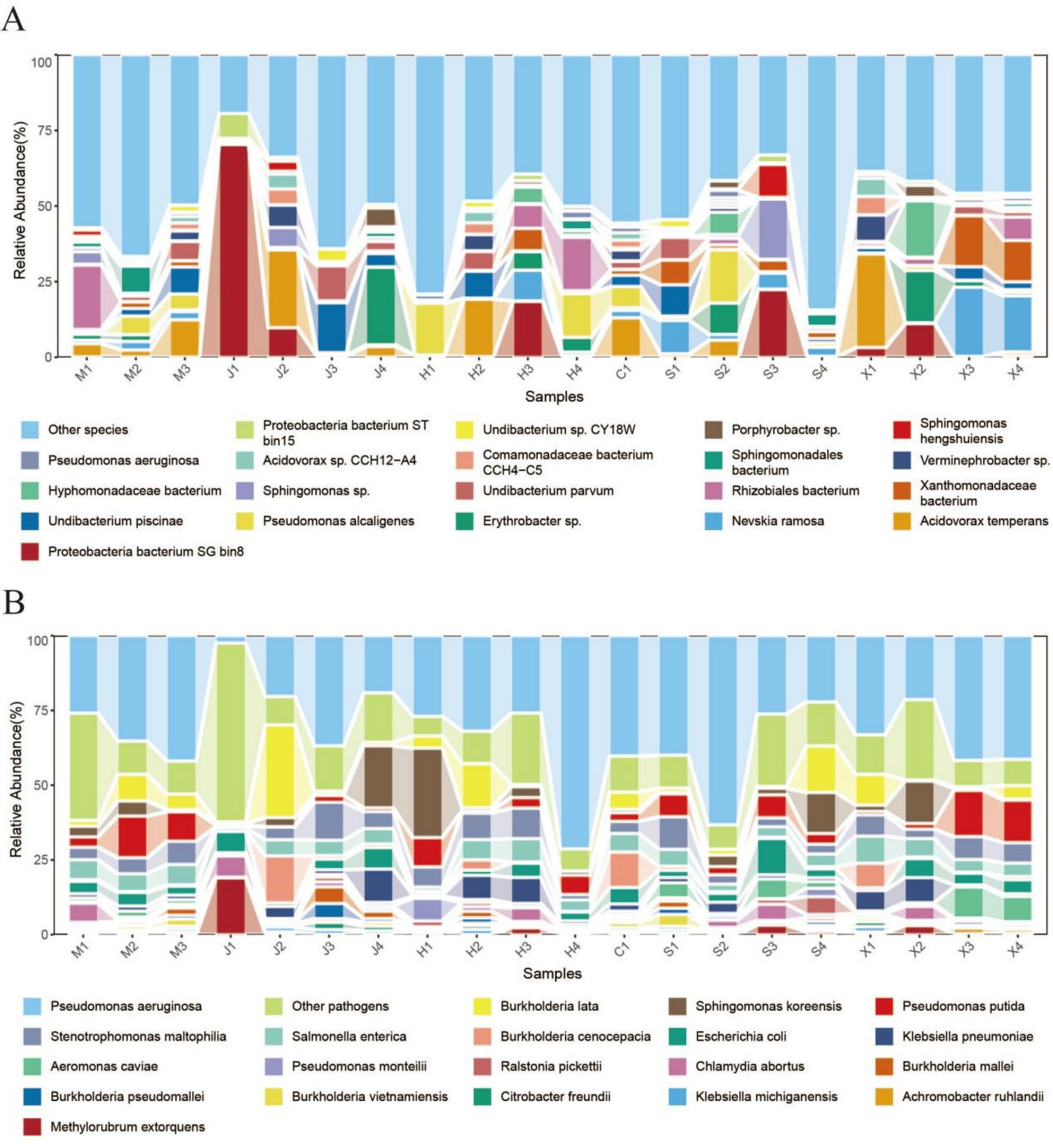
Microbial distribution in the swimming pool water. **A** Stacked bar chart of the top 20 most abundant microbial species; **B** Stacked bar chart of the top 20 most abundant pathogenic microbial species

Metagenomic sequencing identified the presence of 24,035 microbial species in swimming pool water, with the abundance of bacteria (99.46%), followed by archaea (0.29%), viruses (0.20%), and fungi (0.05%). The predominant fungal species identified included *Tilletia caries* (< 0.01%), *Batrachochytrium dendrobatidis* (< 0.01%), *Basidiobolus meristosporus* (< 0.01%), *Rozella allomycis* (< 0.01%), and *Spizellomyces punctatus* (< 0.01%). The five most abundant viruses were uncultured *Caudovirales phage* (0.10%), *Prokaryotic dsDNA virus sp*. (0.02%), *Sphingomonas phage Eidolon* (< 0.01%), *Pseudomonas virus Noxifer* (< 0.01%), and *Myoviridae sp*.(< 0.01%). As shown in Table 4, the dominant phyla in swimming pool water were Pseudomonadota (96.74%), and Actinobacteria (0.57%). The dominant classes were Betaproteobacteria (37.30%), Alphaproteobacteria (31.27%), Gammaproteobacteria (19.00%), and unclassified groups (10.32%). At the order level, the most abundant were Burkholderiales (35.30%), Sphingomonadales (17.30%), unclassified (11.88%), Rhizobiales (8.20%), and Pseudomonadales (7.56%). The most prevalent families were unclassified (19.03%), Oxalobacteraceae (15.02%), Comamonadaceae (14.64%), Sphingomonadaceae (9.59%), and Pseudomonadaceae (7.44%). The dominant microbial genera were unclassified (26.17%), Undibacterium (12.62%), Acidovorax (9.08%), Pseudomonas (7.41%), and Nevskia (4.41%). As shown in Fig. 2A, the top 20 most prevalent microbial species included *Proteobacteria bacterium SG bin8* (6.88%), *Acidovorax temperans* (6.05%), *Nevskia ramosa* (4.22%), *Erythrobacter sp.* (3.77%), and *Pseudomonas alcaligenes* (3.68%). Furthermore, a total of 441 pathogenic microorganisms were identified, including 338 bacteria, 102 fungi, and 1 virus. As shown in Fig. 2B, the top 20 most abundant pathogenic microorganisms included *Pseudomonas aeruginosa* (1.01%), *Burkholderia lata* (0.20%), *Sphingomonas koreensis* (0.18%), *Pseudomonas putida* (0.17%), and *Stenotrophomonas maltophilia* (0.16%). Pathogen biosafety levels (BSLs) were distributed as follows: BSL-1 (97), BSL-2 (321), BSL-3 (23). The top 50 most abundant pathogenic microorganisms are listed in Table 5.

**Table 4:** Dominant 20 microbial taxa in the swimming pool water

Phylum	Abundance (%)	Class	Abundance (%)	Order	Abundance (%)	Family	Abundance (%)	Genus	Abundance (%)
Pseudomonadota	96.74	Betaproteobacteria	37.30	Burkholderiales	35.30	Unclassified	19.03	Unclassified	26.17
Actinobacteria	0.57	Alphaproteobacteria	31.27	Sphingomonadales	17.30	Oxalobacteraceae	15.02	Undibacterium	12.62
unclassified	0.55	Gammaproteobacteria	19.00	unclassified	11.88	Comamonadaceae	14.64	Acidovorax	9.08
Cyanobacteria	0.38	unclassified	10.32	Rhizobiales	8.20	Sphingomonadaceae	9.59	Pseudomonas	7.41
Bacteroidetes	0.29	Actinobacteria	0.54	Pseudomonadales	7.56	Pseudomonadaceae	7.44	Nevskia	4.41
Firmicutes	0.18	Deltaproteobacteria	0.23	Nevskiales	5.42	Erythrobacteraceae	6.29	Erythrobacter	4.04
Uroviricota	0.18	Caudoviricetes	0.18	Xanthomonadales	3.89	Sinobacteraceae	5.37	Sphingomonas	3.84
Acidobacteria	0.15	Bacteroidia	0.11	Rhodobacterales	3.78	Xanthomonadaceae	3.70	Cupriavidus	2.18
Candidatus Melainabacteria	0.15	Bacilli	0.10	Enterobacterales	0.68	Burkholderiaceae	3.53	Porphyrobacter	2.01
Planctomycetes	0.10	Chlamydiia	0.08	Rhodocyclales	0.64	Hyphomonadaceae	2.18	Novosphingobium	1.82
Chlamydiae	0.10	Flavobacteriia	0.07	Nitrosomonadales	0.62	Rhizobiaceae	1.63	Sphingopyxis	1.76
Verrucomicrobia	0.09	Clostridia	0.07	Rhodospirillales	0.59	Rhodobacteraceae	1.56	Verminephrobacter	1.72
Thaumarchaeota	0.06	Hydrogenophilalia	0.06	Caulobacterales	0.51	Bradyrhizobiaceae	1.09	Rhizobium	1.27
Chloroflexi	0.06	Planctomycetia	0.06	Corynebacteriales	0.25	Enterobacteriaceae	0.60	Sphingobium	1.23
Nitrospirae	0.05	Nitrospira	0.05	Neisseriales	0.25	Caulobacteraceae	0.50	Burkholderia	0.57
Spirochaetes	0.02	Oligoflexia	0.05	Micrococcales	0.21	Hyphomicrobiaceae	0.41	Bradyrhizobium	0.46
Gemmatimonadetes	0.02	Epsilonproteobacteria	0.03	Methylococcales	0.20	Alcaligenaceae	0.39	Mitsuaria	0.45
Chlorobi	0.02	Sphingobacteriia	0.03	Caudovirales	0.18	Phyllobacteriaceae	0.35	Massilia	0.44
Euryarchaeota	0.02	Oscillatoriophycideae	0.03	Synechococcales	0.16	Rhodospirillaceae	0.33	Blastomonas	0.41
Deinococcus-Thermus	0.02	Verrucomicrobiae	0.03	Alteromonadales	0.15	Methylobacteriaceae	0.33	Herbaspirillum	0.38

**Table 5:** Detection rates and BSL classifications of the 50 most prevalent pathogenic microorganisms in swimming pool water

Pathogens	Relative abundance (%)	Detection rates (%)	Biosafety level
Pseudomonas aeruginosa	1.01	100	BSL-2
Burkholderia lata	0.20	100	BSL-1
Sphingomonas koreensis	0.18	100	BSL-1
Pseudomonas putida	0.17	100	BSL-2
Stenotrophomonas maltophilia	0.16	100	BSL-2
Salmonella enterica	0.13	100	BSL-2
Burkholderia cenocepacia	0.10	100	BSL-2
Escherichia coli	0.09	100	BSL-2
Klebsiella pneumoniae	0.08	100	BSL-2
Aeromonas caviae	0.06	100	BSL-2
Pseudomonas monteilii	0.04	100	BSL-1
Ralstonia pickettii	0.03	100	BSL-1
Chlamydia abortus	0.03	100	BSL-3
Burkholderia mallei	0.03	100	BSL-3
Burkholderia pseudomallei	0.03	100	BSL-3
Burkholderia vietnamiensis	0.02	100	BSL-2
Citrobacter freundii	0.02	100	BSL-2
Klebsiella michiganensis	0.02	100	BSL-2
Achromobacter ruhlandii	0.02	100	BSL-2
Methylorubrum extorquens	0.02	100	BSL-1
Afipia broomeae	0.02	100	BSL-2
Burkholderia gladioli	0.02	100	BSL-2
Streptococcus pneumoniae	0.01	100	BSL-2
Enterobacter hormaechei	0.01	100	BSL-2
Alcaligenes faecalis	0.01	100	BSL-1
Vibrio vulnificus	0.01	100	BSL-2
Vibrio parahaemolyticus	0.01	100	BSL-2
Acinetobacter baumannii	0.01	100	BSL-2
Chlamydia trachomatis	0.01	100	BSL-2
Brucella suis	0.01	100	BSL-3
Yersinia pestis	0.01	100	BSL-3
Enterobacter cloacae	0.01	100	BSL-3
Enterobacter asburiae	0.01	100	BSL-1
Bordetella pertussis	0.01	100	BSL-2
Neisseria gonorrhoeae	0.01	100	BSL-2
Afipia massiliensis	0.01	100	BSL-2
Vibrio mimicus	0.01	100	BSL-2
Acinetobacter ursingii	0.01	100	BSL-2
Acinetobacter nosocomialis	0.01	100	BSL-2
Bordetella bronchiseptica	0.01	100	BSL-2
Comamonas aquatica	0.01	100	BSL-1
Serratia marcescens	0.01	100	BSL-2
Burkholderia cepacia	0.01	100	BSL-2
Klebsiella aerogenes	0.01	100	BSL-2
Afipia clevelandensis	< 0.01	100	BSL-2
Acinetobacter haemolyticus	< 0.01	100	BSL-2
Burkholderia glumae	< 0.01	95	BSL-1
Enterobacter roggenkampii	< 0.01	100	BSL-2
Afipia birgiae	< 0.01	100	BSL-2
Streptococcus dysgalactiae	< 0.01	100	BSL-2
Staphylococcus aureus	< 0.01	100	BSL-2
Yersinia intermedia	< 0.01	95	BSL-2
Enterococcus faecium	< 0.01	100	BSL-2
Aeromonas veronii	< 0.01	100	BSL-2
Klebsiella oxytoca	< 0.01	90	BSL-2
Aeromonas hydrophila	< 0.01	100	BSL-2
Staphylococcus hominis	< 0.01	100	BSL-2
Achromobacter piechaudii	< 0.01	95	BSL-2
Mycobacterium avium	< 0.01	100	BSL-2
Coxiella burnetii	< 0.01	90	BSL-3
Mycobacterium tuberculosis	< 0.01	100	BSL-3
Rickettsia prowazekii	< 0.01	100	BSL-3
Mycobacterium kansasii	< 0.01	100	BSL-2
Brucella abortus	< 0.01	100	BSL-3
Brucella neotomae	< 0.01	95	BSL-3
Afipia felis	< 0.01	100	BSL-2
Phocaeicola dorei	< 0.01	100	BSL-1
Vibrio furnissii	< 0.01	95	BSL-2
Legionella jordanis	< 0.01	95	BSL-2
Vibrio cholerae	< 0.01	100	BSL-2

### Potential origins of microbial communities in the pool water

**Fig. 3:**
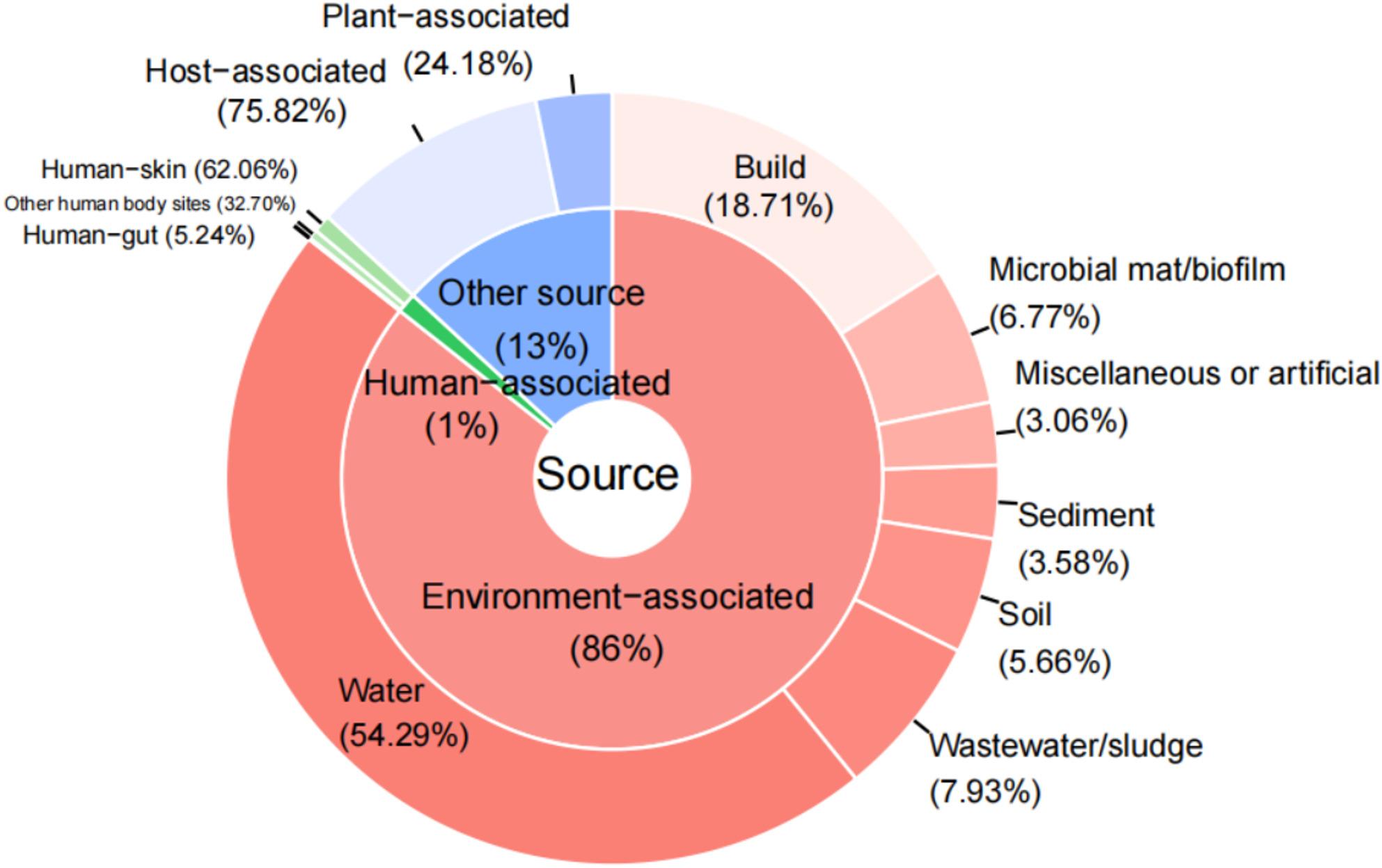
Potential Sources of Microbial Communities in the Pool Water

The composition and putative sources of microbial communities in the swimming pool water are illustrated in Fig. 3. Environmental sources constituted the dominant origin (86.00%) of the pool water microbiome. Within this category, water itself was the primary contributor, followed by built environments, wastewater/sludge, microbial mats/biofilms, soil, sediment, and miscellaneous or artificial sources. Human-associated microorganisms accounted for a smaller but distinct portion (1.00%) of the community, with notable contributions from skin, gut, and other body site microbiota. Plants and animals served as additional reservoirs, contributing the remaining proportion to the pool microbial assemblage.

### Effects of geographical location on microbial composition in the pool water

**Fig. 4:**
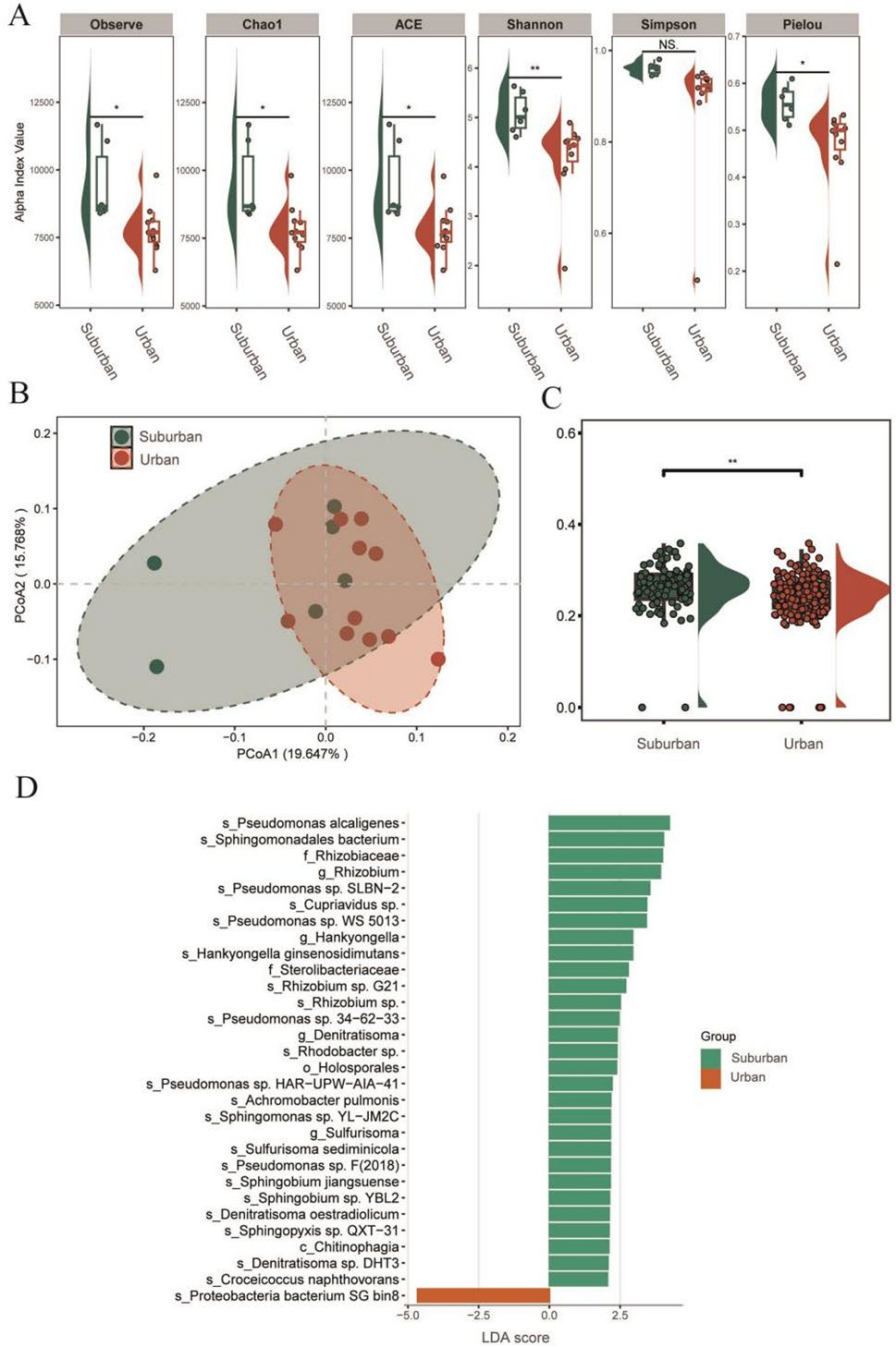
Microbial community variations between swimming pool water collected from urban and suburban pools. **A** *α*-Diversity variations; **B**-**C** *β*-Diversity dissimilarities via PCoA (Bray-Curtis distance); **D** LEfSe-based identification of differentially enriched taxa

Suburban pools exhibited higher Chao1, Shannon, and Simpson indices than urban pools (*P* < 0.05; Fig. 4A). Meanwhile, principal coordinates analysis (PCoA) showed significant *β*-diversity differences between urban and suburban pools (*P* < 0.05), with greater microbial heterogeneity in suburban sites (Fig. 4B-C). LEfSe analysis of the dominant species corroborated these findings, highlighting distinct microbial communities compositions between urban and suburban pools (*P* < 0.05). Specifically, there were 1 dominant class, 1 dominant order, 2 dominant families, 4 dominant genera, and 22 dominant species in suburban pools. Suburban pools were enriched in Chitinophagia class, Holosporales order, Rhizobiaceae and Sterolibacteriaceae family, as well as *Rhizobium*, *Hankyongella*, *Denitratisoma*, *Pseudomonas alcaligenes*, *Sphingomonadales* bacterium, and *Pseudomonas* sp. SLBN − 2, whereas urban pools had higher *Proteobacteria bacterium SG bin8* (Fig. 4D).

### Effects of physicochemical factors on microbial composition in the pool water

**Fig. 5:**
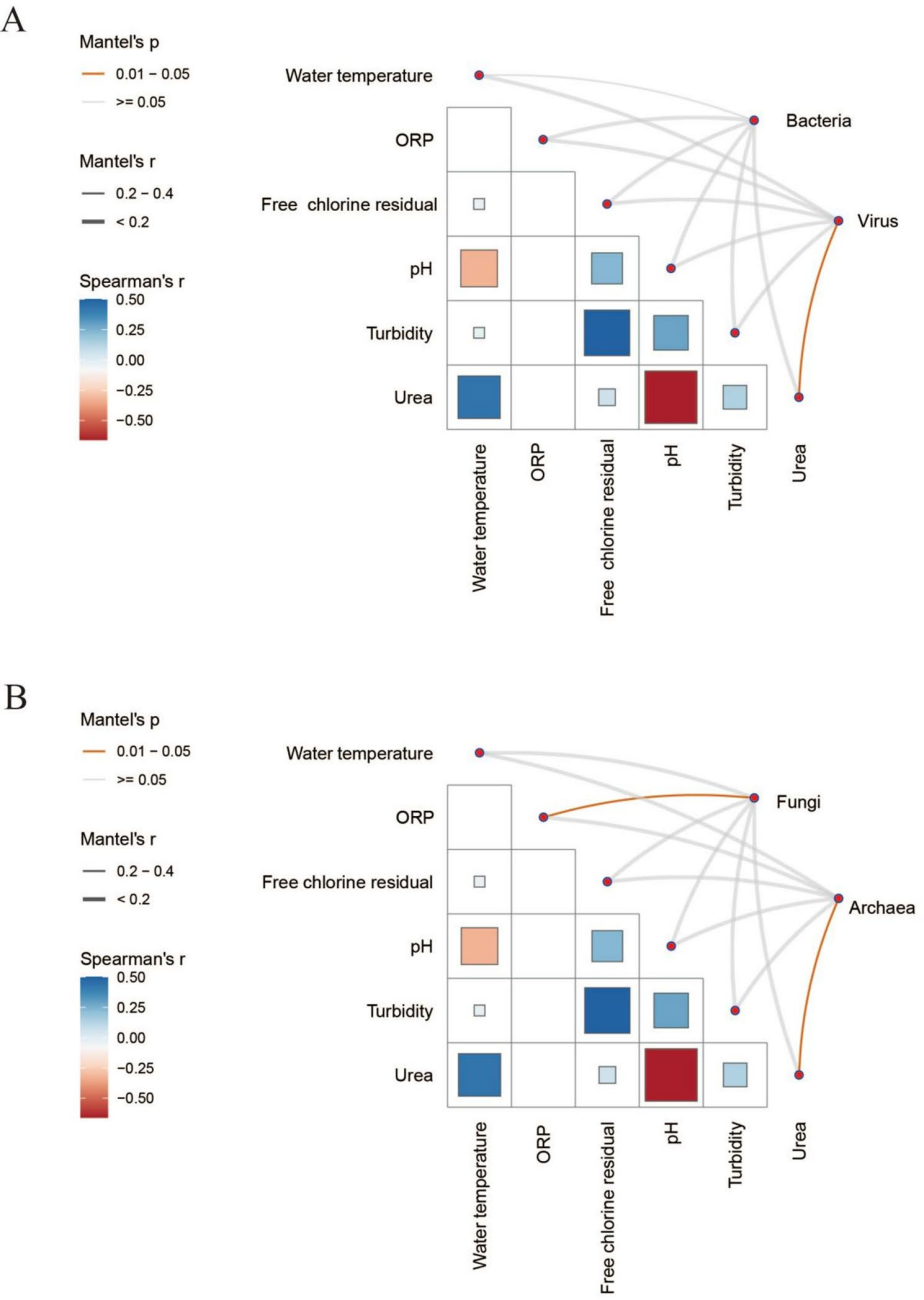
The association between physicochemical factors and microbial composition in swimming pool water. **A** The association between physicochemical factors and the composition of bacteria and virus, **B** The association between physicochemical factors and the composition of fungi and archaea

Spearman correlation analysis identified significant associations between key water quality parameters (Fig. 5). Water temperature was found to be positively correlated with urea (*r* = 0.43, *P* < 0.05). The pH of pool water was negatively correlated with urea concentration (*r* = − 0.66, *P* < 0.05). A positive correlation was also observed between the free residual chlorine concentration and turbidity (*r* = 0.50, *P* < 0.05). Moreover, the Mantel test demonstrated that the distribution of viruses and archaea in swimming pool water was positively correlated with urea concentration in Fig. 5 (*r* = 0.31, *P* < 0.05; *r* = 0.25, *P* < 0.05). Similarly, a positive correlation between the distribution of fungi and oxidation-reduction potential was detected (*r* = 0.25, *P* < 0.05). The distribution of bacteria was not statistically correlated with physicochemical factors in swimming pool water (*P* ≥ 0.05).

## Discussion

Conventionally, researchers have predominantly employed PCR techniques and isolation culture methods to identify pathogens in swimming pool water. However, these conventional approaches are inherently limited by their inability to achieve comprehensive detection of diverse pathogenic microorganisms. In contrast, metagenomic sequencing technology capable of identifying both known and novel microbial taxa has emerged as a transformative tool in public health research. This study employs metagenomic sequencing to conduct an in-depth characterization of microbial communities in public pool water, revealing important insights into the microbial diversity, the effects of geographical location and physicochemical factors on microbial composition. These findings have substantial implications for understanding the complex microbial ecosystems of swimming pools and improving the Public health risk management in swimming pool.

This study identified 24,035 microbial species in swimming pool water, a significantly higher number than the 9,475 species identified in our previous research on subway air microorganisms [44]. This difference likely stems from the more hospitable conditions that aquatic environments provide for microbial proliferation compared to air. The microbial community in swimming pool water was dominated by the Pseudomonadota phylum (96.74%), followed by Actinobacteria phylum (0.57%), a taxonomic distribution consistent with previous reports on drinking water microbiome [45–47]. Moreover, the predominant bacterial phyla in soil are also Proteobacteria and Actinobacteria [48]. However, microbiomes in air exhibited some differences with that in water. Airborne bacteria are predominantly composed of Pseudomonadota, with a substantial proportion of Bacillota (formerly Firmicutes) [49, 50]. At the class level, Betaproteobacteria (37.30%) emerged as the most abundant taxon in pool water. This finding aligns with existing researches, as Betaproteobacteria includes numerous ubiquitous genera commonly detected in drinking water systems [51]. Thus, these results indicate that the microbial community structure in swimming pools closely mirrors that of natural and engineered aquatic environment, suggesting that pool microbiota are primarily originate from environment sources [52].

Moreover, a total of 441 pathogenic microorganisms were detected in public swimming pool water, including the most abundant opportunistic pathogens, such as *Pseudomonas aeruginosa* (ranked 1st), *Acinetobacter baumannii* (8th), and *Staphylococcus aureus* (17th) [53, 54]. These bacteria, common in both environment and human, are generally non-pathogenic in hosts with robust immune systems. However, individuals with skin injuries or compromised immune systems are more susceptible to infections [55]. Therefore, these individuals should avoid swimming in public swimming pools. The detection of Mycobacterium spp, known etiological agents of cutaneous non-tuberculous granulomatous infections aligns with previous epidemiological findings [23, 56]. Similarly, the presence of multidrug-resistant organisms like Stenotrophomonas maltophilia (0.16%) raises concerns about potential transmission of antibiotic resistance genes in pool environments. At the order level, the most abundant were Burkholderiales (35.30%) in public swimming pool water. This order includes the most dangerous bacterial that may be involved in cystic fibrosis [57]. Applying our conservative filtering criteria, we detected sequence reads matching several BSL-3 pathogens in swimming pool water. Given the use of chlorine disinfection, these sequences likely originate from nonviable organisms or free DNA rather than from active pathogens. Nevertheless, the presence of such genetic remnants implies that insufficient disinfection may permit the survival of viable pathogens, thereby posing an infection risk to swimmers [7, 58].

Additionally, the substantial proportion of unclassified taxa at all taxonomic levels (ranging from 10.32% to 26.17%) highlights significant gaps in current microbial databases and complex microbial ecosystems of swimming pools. This finding also suggests that swimming pools may harbor many previously unknown microbial species, potentially including novel pathogens or organisms with unique metabolic capabilities. Therefore, expanding and refining microbial genomic databases represents an urgent priority for public health microbiology.

Analysis of microbial origins revealed that water, buildings and microbial mat/biofilm were the primary contributors to the pool’s microbial community. Therefore, to minimize microbial introduction, it is critical to introduce clean source water and perform regular scrubbing of the pool. Our findings indicate that suburban swimming pools, which exhibited higher microbial species diversity and a substantially larger number of dominant species (29 vs. 1) than urban pools, should be considered a key target for enhanced surveillance by health monitoring departments. Further investigation is required to ascertain the key factors contributing to these outcomes, such as, environmental conditions, swimmers demographics, and water quality management.

China has established a regulatory framework for public swimming pool water quality, primarily based on the *Regulations on Health Administration in Public Places* (revised in 2024) and the national standard *Hygienic indicators and limits for public places* (GB 37488 − 2019) (currently under revision) [43]. The framework operates under a health permit system, requiring swimming venues to obtain approval from health authorities prior to operation and mandating that all staff in direct contact with patrons hold valid health certificates. GB 37,488 − 2019 stipulates mandatory limits for key physicochemical and microbiological parameters, mainly including free residual chlorine, pH, turbidity, urea, total bacterial count, and coliforms. Regulatory enforcement is led by health authorities through a “dual-random, one-open” inspection mechanism, whereby inspection targets and personnel are randomly selected and results are publicly disclosed to ensure transparency and facilitate consumer oversight. In this study, the water quality of 20 swimming pools was assessed against the requirements of GB 37,488 − 2019. The compliance rates for water temperature, ORP, free residual chlorine, pH, urea, and turbidity were 70.00%, 75.00%, 75.00%, 85.00%, 85.00%, and 100.00%, respectively, indicating an overall satisfactory water quality across the sampled pools. To assess the influence of water quality on microbial composition, we employed the Mantel test, a nonparametric statistical method well-suited for evaluate correlations between independent distance matrices. Notably, the distribution of viruses in swimming pool water was found positively correlated with the concentration of urea. This association may be attributed to urea-induced depletion of free chlorine compromises disinfection efficacy and increased viral introduction and urea concentrations followed by bather load. This founding implies that urea concentration monitoring could serve as a potential indicator for viral contamination risk.

Under the current regulatory framework for public swimming pools in China, various pathogenic microorganisms have still been detected in pool water. Notably, urea has emerged as a water quality parameter significantly associated with both viruses and archaea. This finding underscores the critical importance of continuous monitoring and control of urea levels for the effective management of pathogens in swimming pool water. To mitigate the risk of pathogen contamination and increase public awareness of recreational water illnesses, public health campaigns emphasizing the importance of pre-swim showers and the hazards of urination in the pool are necessary.

Although this study provides valuable insights, several limitations should be acknowledged. First, the single time-point sampling precludes the assessment of temporal dynamics of microbial community composition in swimming pools. The application of metagenomic sequencing has effectively revealed all microorganisms in swimming pool water, but cannot distinguish viable from non-viable microorganisms. These limitations highlight important directions for future research.

## Conclusion

This study, utilizing metagenomic sequencing technology, revealed a range of microorganisms with varying pathogenic potentials in swimming pools. Furthermore, a positive correlation was observed between the concentration of urea in swimming pool water and the distribution of viruses. The activity levels and quantities of pathogenic microorganisms in swimming pool water cannot be directly measured, but inadequate disinfection may pose a significant risk of disease transmission. To mitigate such risks, swimmers are encouraged to follow the principles of civilized swimming, including pre-swim showering and avoidance of urination in the pool. Additionally, it is essential for swimming facility operators to strictly adhere to standardized hygiene protocols. This includes introducing clean source water, performing regular scrubbing of the pool and closely monitoring urea concentrations to minimize public health risks.

## Data Availability

The sequence reads from all samples collected during this study were deposited in the NCBI data bank (BioProject accession number PRJNA1345502).
